# Ten–Second Electrophysiology: Evaluation of the 3DEP Platform for high-speed, high-accuracy cell analysis

**DOI:** 10.1038/s41598-019-55579-9

**Published:** 2019-12-16

**Authors:** Kai F. Hoettges, Erin A. Henslee, Ruth M. Torcal Serrano, Rita I. Jabr, Rula G. Abdallat, Andrew D. Beale, Abdul Waheed, Patrizia Camelliti, Christopher H. Fry, Daan R. van der Veen, Fatima H. Labeed, Michael P. Hughes

**Affiliations:** 1 Centre for Biomedical Engineering, Department of Mechanical Engineering Sciences, University of Surrey, Guildford, Surrey GU2 7XH UK; 2 School of Biosciences and Medicine, University of Surrey, Guildford, Surrey GU2 7XH UK; 3 Department of Biomedical Engineering, Faculty of Engineering, The Hashemite University, PO Box 330127, Zarqa, 13133 Jordan; 4 Present Address: Department of Electrical Engineering and Electronics, University of Liverpool, Brownlow Hill, Liverpool L69 3GJ UK; 5 Present Address: Department of Engineering, Wake Forest University, Wake Downtown, Winston-Salem NC 27109 USA; 6 Present Address: MRC Laboratory for Molecular Biology, Francis Crick Avenue, Cambridge, CB2 0QH UK; 7 Present Address: School of Physiology, Pharmacology & Neuroscience, University of Bristol, University Walk, Bristol, BS8 1TD UK

**Keywords:** Biomedical engineering, Circadian rhythms, Membrane trafficking, Membrane biophysics

## Abstract

Electrical correlates of the physiological state of a cell, such as membrane conductance and capacitance, as well as cytoplasm conductivity, contain vital information about cellular function, ion transport across the membrane, and propagation of electrical signals. They are, however, difficult to measure; gold-standard techniques are typically unable to measure more than a few cells per day, making widespread adoption difficult and limiting statistical reproducibility. We have developed a dielectrophoretic platform using a disposable 3D electrode geometry that accurately (r^2^ > 0.99) measures mean electrical properties of populations of ~20,000 cells, by taking parallel ensemble measurements of cells at 20 frequencies up to 45 MHz, in (typically) ten seconds. This allows acquisition of ultra-high-resolution (100-point) DEP spectra in under two minutes. Data acquired from a wide range of cells – from platelets to large cardiac cells - benchmark well with patch-clamp-data. These advantages are collectively demonstrated in a longitudinal (same-animal) study of rapidly-changing phenomena such as ultradian (2–3 hour) rhythmicity in whole blood samples of the common vole (*Microtus arvalis*), taken from 10 µl tail-nick blood samples and avoiding sacrifice of the animal that is typically required in these studies.

## Introduction

Since the work of Galvani in the 1790s it has been known that the function of many biological tissues is related to electricity, and in particular to the transmembrane movement of electrical charge as ions, or changes to electrostatic potential. The concept of a cell membrane as a high, but variable, resistance barrier with capacitive properties was subsequently developed by measurement of the frequency-dependent impedance properties of many plant and animal tissues, culminating in the seminal work by Hodgkin, Huxley and Katz^1^. Subsequently, these electrophysiological principles have been used to address a number of questions including transmission of information in the nervous system and excitation-contraction/secretion coupling^2^. More recently, changes in membrane potential due to disturbances of cytoplasmic ion composition have been associated with cancer phenotypes^3^. Even in non-excitable cells, key cellular processes are mediated by ion channel activity, such as Ca^2+^ and K^+^ movements underpinning apoptosis^4^, or characteristic changes of membrane impedance that occur during cellular differentiation and transformation^5,6^.

Electrophysiological methods generally use intracellular electrodes to measure changes in transmembrane potential or impedance, or ion flux under voltage-clamp. This approach was hugely advanced by the development of patch-clamp techniques that allowed more accurate measurements with cells less accessible to previous conventional configurations^7^. Patch-clamp uses a micropipette to form a high resistance (GΩ) seal between the pipette and the cell to allow the time- and voltage-dependent electrical properties of a patch of cell membrane, or that of the whole cell, to be measured directly. The technique remains in widespread use, but is slow, requires skilled operators, and is reliant upon expensive equipment that only records from one cell at a time. Automated microfluidic patch-clamp instruments^8^ have recently become available. These can perform simultaneous tests on 20 or more cells, using disposable chips, but the cost of such apparatus can be prohibitive. An alternative method for measuring changes of membrane potential involves the use of lipid-soluble fluorescent probes whose excitation/emission characteristics vary as a function of membrane potential^9^. Such methods are limited, however, since they have small dynamic range, poor quantum efficiency, they cannot measure the resistive and capacitive properties of cells, and can interfere with normal membrane function. Taken together, these factors make assessment of cellular electrical properties at scale and speed highly difficult to achieve and have prevented such measures being adopted widely across cell biology.

Other methods of assessing electrophysiological state exploit the interaction of cells with electrical fields to measure their passive and active electrical properties. Such methods can be classified according to whether current is passed through the cell (and surrounding medium) in order to determine its impedance; or how the cell interacts with the field is measured. The former approach is best embodied by impedance spectroscopy^10–13^, whereby the properties of cells are elucidated by direct measurement of impedance through the use of microengineered electrodes and microfluidics. Such devices allow single-cell analysis of properties, but at a cost of throughput (below 100 s^−1^, often much slower) and device cost.

The second approach is to study the forces generated in individual cells due to their interaction with electric fields. We have developed a novel instrument for the ensemble analysis of cellular electrical properties through dielectrophoresis (DEP), the movement of particles within non-uniform electric fields. During DEP, cells are subject to an oscillating electric field and, as a consequence of the transient electric dipoles that arise will either be attracted to, or repelled from, areas of high electric field gradient as a function of the frequency of the applied field^14^. Every membrane-bound cell or sub-cellular particle has dielectric properties such as surface charge and membrane capacitance, as well as the resistive properties of the membrane and cell/particle interior (cytoplasm). Together these fundamental properties describe both the biological state of the cell and determine the response of a cell during DEP experiments^15^. The values of these electrical parameters may be robustly interpolated from dielectrophoretic responses of cells: measured as the way in which the magnitude and direction of DEP responses vary as a function of frequency. The resultant “DEP-spectrum” describes the electrical properties of the cells in a rapid, cost-effective and non-invasive way^16,17^.

DEP has been used to measure electrical properties of excitable cells and benchmarks well against other, validated methods^18^. DEP-spectra have also successfully defined biomarkers for stem cell differentiation^5,19^, cancer^20,21^, drug interactions^22–24^ and circadian biology^25,26^. However, although DEP is most effective for measuring cell electrical properties, its adoption as a method in general cell biology has been limited by electrode technology and the challenges associated with robust data analysis. Hitherto, electrodes generally consisted of two-dimensional structures etched onto glass slides and observed with a camera. Such systems characterised relatively low cell numbers, and were highly complex to operate; measurements were limited to low-conductivity media, whilst achieving a full DEP-spectrum was time consuming. This created pressure to produce low-resolution spectra with few points, and hence introduced error when fitting biophysical models to data in order to extract parameters^27^.

To better realise DEP as an analytical technique, we developed a novel approach to the construction and embodiment of three-dimensional electrodes drilled from laminates of copper and glass fibre reinforced epoxy resin and then gold-plated, for stability and biocompatibility^17^. These “DEP-wells” have annular electrodes along the length of the side wall, reducing DEP-induced cell movement to 1-dimension: cells move towards or away from the line passing through the centre of the well at a rate proportional to the DEP force they experience. Whilst the technique lacks single-cell precision compared with impedance cytometry, it more than compensates for this though speed, throughput and ease of use. Previous systems described in the literature used simple single-well systems to characterise cells one frequency at a time and at up to two minutes per measurement, meaning a complete spectrum might take an hour. In this paper we describe the performance of the first commercial DEP cytometry platform, the DEPtech 3DEP (Labtech, Heathfield, UK), and show how this platform significantly improves on previous DEP-well performance through parallel signal generation and improved optics and signal processing^28^. This permits simultaneous characterisation of thousands of cells, acquiring data at high speed, and potentially making measurements in cell suspensions of high electrical conductivity, allowing measurements of electrophysiological phenomena which are difficult to achieve by other means.

## System Description

### 3DEP analysis

**Figure 1:**
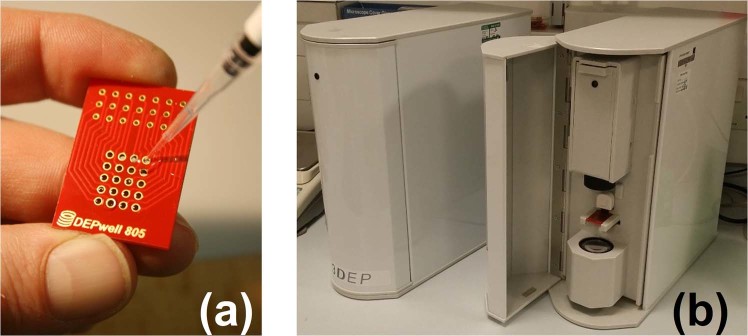
The 3DEP system comprises two components, a chip (**a**) which is inserted into an instrument unit. The chip measures approximately 20 × 30 mm. (**b**) shown here with door closed (left) and open to show the optical path and chip (right). The chip contains 20 wells, transparent along their central axis and joined by a gasket on the underside to allow all 20 wells to be filled simultaneously. When inserted in the instrument, each well is connected to a signal generator providing up to 20 V_pp_ and as high as 45 MHz; with all 20 wells energised with different frequencies a spectrum can be obtained in ten seconds. The instrument also contains control circuitry and an optical system for data acquisition.

**Figure 2:**
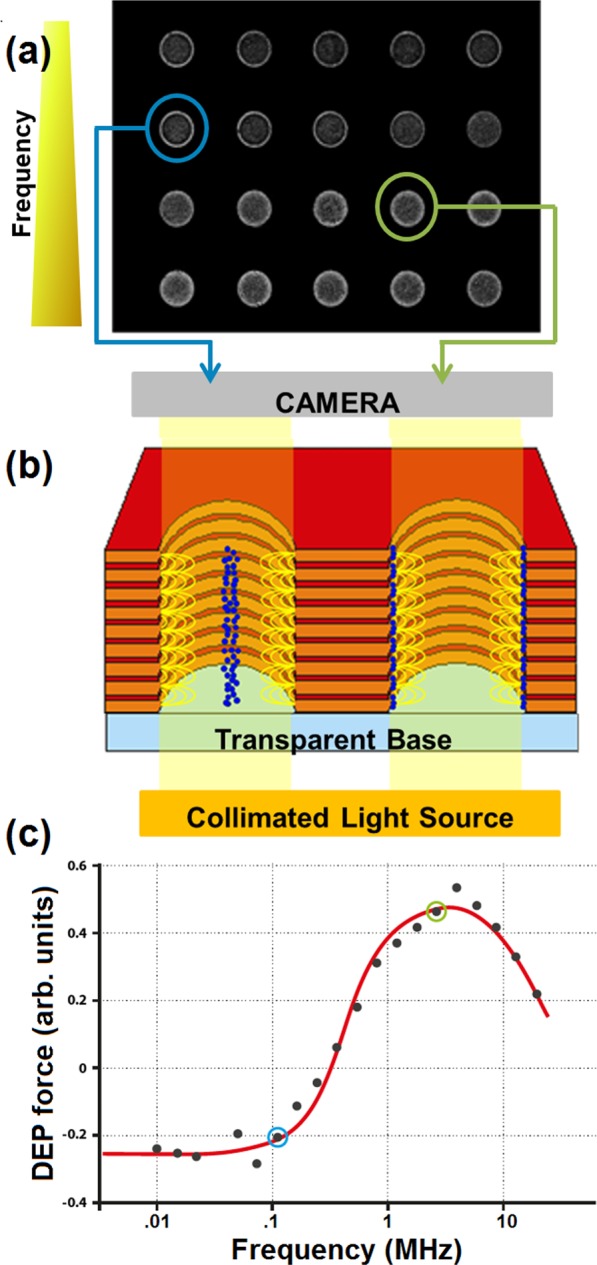
Data acquisition sequence. An image of the chip is taken every second, beginning the instant before the chip is energised. Image (**a**) shows a chip loaded with red blood cells, 10 seconds after the field is applied; frequency increases left-to-right along each row, and from top row to bottom row. Cells move towards or away from the electrodes at the well walls, termed positive and negative DEP respectively, as seen in the left and right circled wells in (**a**) and shown schematically in (**b**). Analysis of the DEP behaviour at different frequencies allows determination of the membrane conductance and capacitance, and cytoplasmic conductance; the spectrum shown in (**c**) is that taken from the chip image in (**a**), with the left and right highlighted dots in (**c**) corresponding to the highlighted wells in image (**a**). The Clausius-Mossotti model (line) fitted to the data points has an r^2^ correlation of 0.995.

The system comprises a chip (Fig. 1a) inserted into a reader (Fig. 1b) containing twenty independently-controlled DDS-based signal generators, each capable of delivering 20 V_pp_ at frequencies up to 45 MHz. When the electric field is applied, cells redistribute within the well according to their DEP response at that frequency, and changes in light absorption from a number of concentric annular regions is monitored to track the redistribution of cells. When the electrodes within the chip are energised, the dielectrophoretic force causes cells to move either towards or away from the well edges, meaning that the change in light absorbance through the well allows determination of cell polarisability at that frequency. Since the wells are axisymmetric, the light intensity can be reduced to a one-dimensional axial value. Simultaneous energising of 20 wells with different frequencies permit parallel acquisition of 20-point spectra and reduce the time to acquire a full DEP-spectrum from hours to seconds (Fig. 2); spectra are typically acquired in 10 seconds, though larger mammalian cells can produce high-quality data in as little as 3 seconds. Extension of the upper frequency limit to 45 MHz is significant as it permits observation of almost all of the high-frequency dispersion, allowing more accurate determination of the cytoplasmic electrical properties.

### The 3DEP electrode

The 3D chip represents a significant advance on devices developed previously^17,29–31^. Chips comprise alternating layers of conductors (copper, 70 µm thick) and insulators (glass fibre reinforced epoxy (FR4), 150 µm thick) which, when drilled, form wells (1 mm diameter) with electrode “rings” stacked on the side wall. The exposed electrode surfaces are gold-plated to ensure biocompatibility, and each well is served by a separate set of power lines, allowing independent signals to be sent to each well. After fabrication, a gasket and glass window are affixed to the base. The gasket hydraulically connects the well interiors, allowing cell solution to pass between the wells. As a consequence, a 20-well chip is filled by adding approximately 80 µl of solution through a single well. A cover slip is placed over the filled wells before being placed in the instrument. Wells are of similar size and pitch to those found on high-throughput well systems, so that for example, a multi-sample system based on a 1536-well plate would use conventional liquid handling robots with little or no adaptation required. Cells are typically loaded at a concentration of *circa* 10^6^ ml^−1^, and with a total chip volume of 80 µl the total cell number per well is approximately 1000; we have found empirically that DEP-well electrodes can produce modellable spectra from concentrations below 10^4^ ml^−1^ to over 10^8^ ml^−1^, but that noise levels increase below 10^6^ ml^−1^, making this the optimum combination of low cell requirements and low noise.

### The 3DEP platform

**Figure 3:**
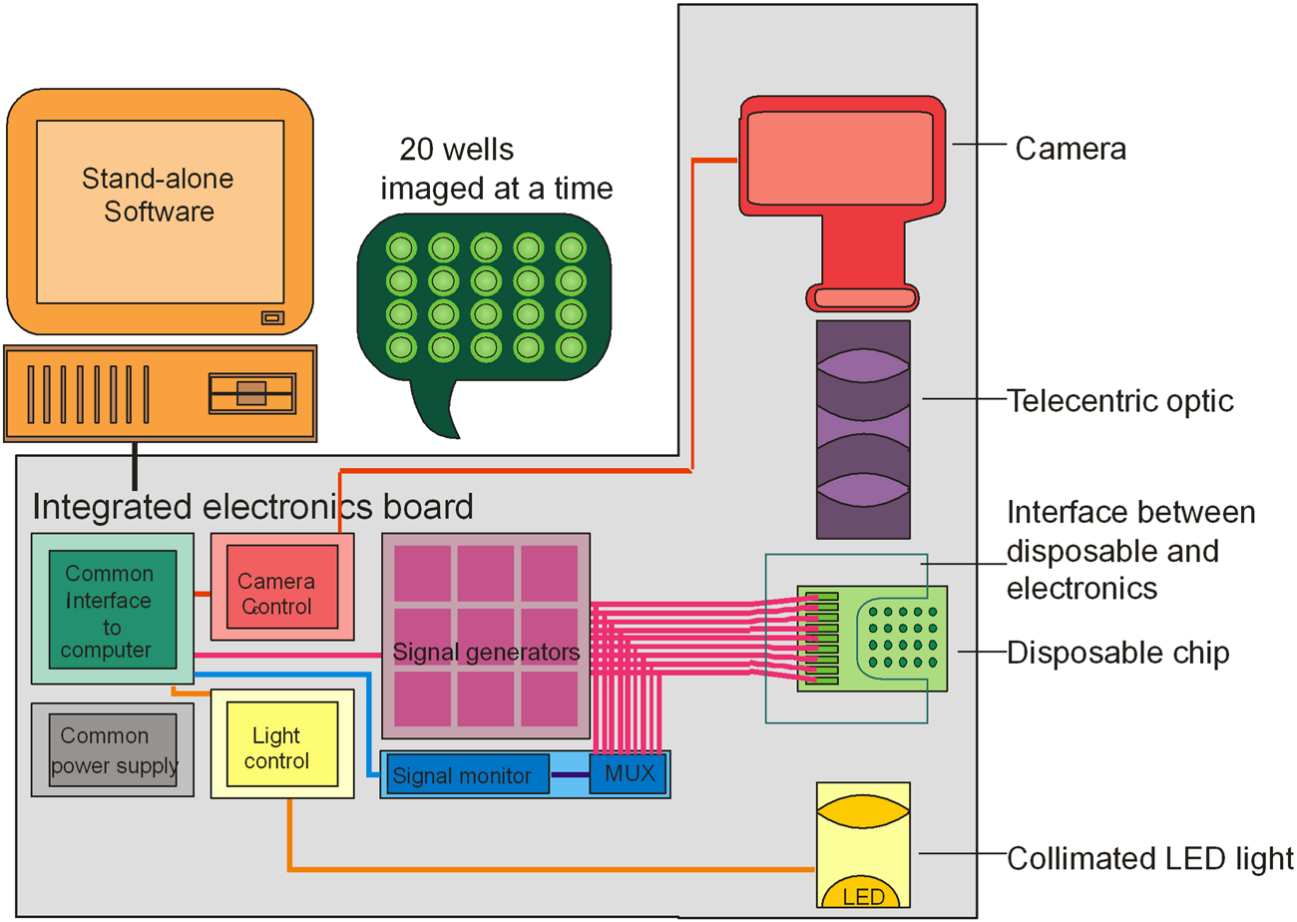
Schematic of the 3DEP reader platform. A chip containing 20 wells (right hand side of image) is connected via an interface to 20 independent signal generators capable of 45 MHz at 20 V_pp_. A control board in the instrument controls the generators and light source and also acquires instrument status information. Camera signals are acquired via a bi-telecentric lens and sent via the unit to a computer, where the analysis is performed.

The system is summarised in Fig. 3. To run an experiment, the chip containing the cell suspension is inserted into a custom, zero-insertion force mount that connects the wells to the signal generators and locks it in place immediately prior to each recording. The chip is then illuminated using a collimated light source and monitored using a CMOS camera fitted with a bi-telecentric optic, allowing the monitoring of all 20 wells simultaneously. During the recording, the wells are energised with 20 user-assignable frequencies, typically selected to cover approximately five points per decade from 1 kHz to 45 MHz. Motion of particles tracked across the wells, using an image analysis algorithm on the host PC. Light absorbance is monitored across ten concentric rings inside each well, and the rate of change in light intensity within these rings is determined and scaled to relative area^29,32^. This parameter is proportional to the Clausius-Mossotti factor, a mathematical relationship for the relative polarisability of the suspended particles which is based, in this instance, on the dielectric properties of cytoplasm, membrane and suspending medium and on the applied frequency^14,27^.

Once the chip is filled and locked into the reader and the device door closed to reduce interference, the system follows a one-click automated process of activating the optical path, signal generators and analysis software. Images of the well are taken once per second, up to a user-defined time limit; theoretical analysis^29^ indicates that the system results are linear up to 10 seconds, which is used as the standard analysis period. Longer periods can be used for a greater signal magnitude where there is noise in the data, for example when the cell concentration is low; however, prolonged run times lead to a less linear response.

The impact on cells is low. The only requirement for cells analysed in the present system is that they are in suspension, which may require detachment. Cells need be present in this medium for no longer than the wash cycle and the time required to take a measurement; a total of approximately fifteen minutes. During this time, cells are exposed to a low-intensity electric field for typically ten seconds. Following measurement, cells will have been exposed to few hazards, and can be recovered and re-cultured provided the measurement is taken under sterile conditions. Recovery is simple; after careful removal of the cover slip, the use of a syringe or micropipette allows the recovery of typically 70% of the cells inserted in the device. Cells can subsequently be centrifuged and resuspended in their typical culture media and re-plated.

## Materials and Methods

### Preparation of cells

#### Cultured cells

The murine cardiomyocyte line HL-1 (kindly donated by Prof W. Claycomb, Louisiana State University), human myelogenous leukaemia line K562, human breast cancer line HeLa and Jurkat human T-lymphocyte line (all purchased from ATCC Cells, UK) were cultured under standard conditions. Prior to experimentation, cells were centrifuged at room temperature at 259 *g* for 6 minutes. The supernatant was removed and the pellets were resuspended in iso-osmotic medium consisting of 8.5% (w/v) sucrose, 0.5% (w/v) dextrose, 100 µM CaCl_2_ and 250 µM MgCl_2_. The medium conductivity was adjusted using PBS to 10 mS.m^−1^ (typically ca. 0.7% by volume) The conductivity was verified using a Jenway conductivity meter (VWR Jencons, Leicestershire, UK). The final cell population was counted using a haemocytometer and adjusted for DEP measurements to 1 × 10^6^ cells ml^−1^ (±15%).

#### Human RBCs and platelets

To obtain human RBCs, anticoagulant-treated whole blood (lithium heparin Vacutainer, BD Biosciences, Franklin Lakes, NJ) was mixed 1:3 with phosphate-buffered saline (Sigma Aldrich), layered onto a density gradient medium Histopaque-1077 and centrifuged at 480 rpm for 30 min at room temperature. The cell pellet was resuspended into PBS to remove non-erythrocytic cells, and centrifuged again at 1270 rpm for 15 min^33^ and the pellet suspended into a DEP medium as described in section 3.1.1, but adjusted to a final conductivity of 43 mS.m^−1^.

Platelet-rich plasma was isolated from the fractionated whole blood sample following the 30-minute centrifuge described above. To avoid platelet activation, further centrifugation was not performed, and only minimal (<1%) RBC contamination was observed. Care was taken to gently pipette the platelet layer which formed on top of the RBC pellet and leucocyte layer. For experiments, 10 µl was resuspended in 5 ml DEP medium and adjusted to 43mS.m^−1^, whilst 200, 400 and 800 m.Sm^−1^ media were achieved by mixing platelet-rich plasma (ca. 1500 mS.m^−1^) and 43 mS.m^−1^ DEP medium at different ratios. Studies were conducted in concordance with the principles of the Declaration of Helsinki; the study received approval from the Ethics Committee of the University of Surrey.

#### Whole vole blood

The vole (*Microtus arvalis*) naturally expresses short (ultradian) feeding-fasting cycles and sleep-wake cycles with a period around 2 hours, whilst being housed under a diurnal light-dark cycle^34,35^. Voles were housed under a 12-hour light, 12-hour dark cycles, for a period of 10 days, with *ad libitum* access to water and food. Behavioural activity was monitored using Passive Infrared motion detection (ClockLab, Actimetrics, Il, USA), confirming ultradian behavioural activity. At the onset of lights on (known as Zeitgeber Time 0; ZT0) on day 10, tail blood samples (10 µl) were collected using minimally invasive tail pricks every 30 minutes, for a period of 4 hours. Whole blood samples were immediately diluted in 5 ml of 10 mS.m^−1^ DEP medium (each sample measured for final conductivity) and analysed within 15 minutes. The extracted electrophysiological parameters were then fitted with a standard cosine curve, with the period fixed to that of the behavioural activity cycles during the preceding 10 days, and phase, mean estimating statistic of rhythm (mesor) and amplitude left to fit as free parameters. All vole work was approved, and carried out in accordance with the guidelines and regulations of the UK Home Office.

### DEP experiments and analysis

Approximately 80 µl of cell suspension was injected into the DEP well chip, and a cover glass placed on top in order to avoid the formation of a meniscus, due to surface tension, which could interfere with the measurement of light intensity changes. The chip was inserted into the chip holder of the 3DEP and the door was closed in order to prevent interference from ambient room lighting. All cells were analysed for 10 seconds except for platelets, which were energised for 30 seconds owing to their smaller size requiring a larger signal. We have studied the 3DEP response across a range of particle concentrations and sizes; in general, for mammalian cells 1e6/ml offers the optimum combination of low noise and low cell requirements. We have obtained spectra at concentrations an order of magnitude below this, and two orders above; at lower concentrations noise increases due to a lack of cells interfering with the light beam, whilst concentrations above 1e7/ml reduces the light passing through the well and again increases noise. Smaller particles such as bacteria require higher concentrations than this (typically by x10), but are not considered here.

An aliquot of each cell sample in DEP medium was transferred onto a haemocytometer. Pictures of the cells dispersed over the grid portion of the haemocytometer were taken in order to measure their radii using Image-J software (National Institute of Health, Maryland, US). The scale was calibrated by measuring the number of pixels across a line of the grid of known length. The cell diameter of cells was then measured by placing a horizontal line across the centre of each cell. One hundred cells were measured for each sample. The distribution of cell size was analysed using statistical analysis software SPSS version 20 (SPSS Inc. Chicago, IL).

Post DEP viability was assessed using a LIVE/DEAD viability/cytotoxicity kit for mammalian cells (Sigma-Aldrich, UK). Cells were washed of the serum enriched media with PBS and then incubated for 15 minutes with 200 µL PBS containing 2 μl.ml^−1^ calcein-AM and 6 μl.ml^−1^ propidium iodide (PI). Calcein-AM is enzymatically converted to green fluorescent calcein in living cells. PI is a nuclein staining dye which cannot pass through a viable cell membrane. It reaches the nucleus by passing through disordered areas of dead cell membrane, and upon nucleic acid-binding produces a red fluorescence.

### Data extraction

Cell electrophysiological properties were extracted by fitting a mathematical model of the Clausius-Mossotti (C-M) factor to the data^27^. This describes the frequency-dependent polarizability of a lossy dielectric (that is, having both resistance and capacitance) spheroids in a lossy dielectric medium, and can be adapted to examine spheroids comprising multiple concentric “shells” around a central “core”. Since the use of higher numbers of cells produces more unknowns than measurable variables, we use a single-shell model to describe the cell membrane and cytoplasm. Where heterogeneous cells are analysed, the resultant DEP spectrum comprises the superposition of the separate C-M factors of different subpopulations^16^; this has been evaluated with cells of similar size (such as live and dead yeast cells)^17^ or different size (such as apoptotic cells and apoptotic bodies)^22^ with good accuracy in discriminating up to four subpopulations; however, in the present work only homogeneous populations were examined. Fitting between the model and data was performed using a Levenberg–Marquardt^28^ best-fit algorithm.

Analysing a large number of cells ensures a high signal-to-noise ratio; spectra from cells such as erythrocytes, regularly fit with a Pearson correlation coefficient r^2^ > 0.99 from a single measurement. Where the number of cells is low, performing several technical replicates yields fits to their collective frequency-response of a similar quality. As the system tracks changes in cell distribution within the wells by monitoring optical absorbance, it is able to observe any particle which interferes with the light beam; it can be used for large cells such as cardiomyocytes. However, it is equally effective for measurement of small cells such as erythrocytes, or even micron-scale entities such as platelets, bacteria or apoptotic bodies, which are difficult or even impossible to analyse using conventional electrophysiological assays^32^.

## Results and Discussion

### Ultra-high-resolution DEP spectra

**Figure 4:**
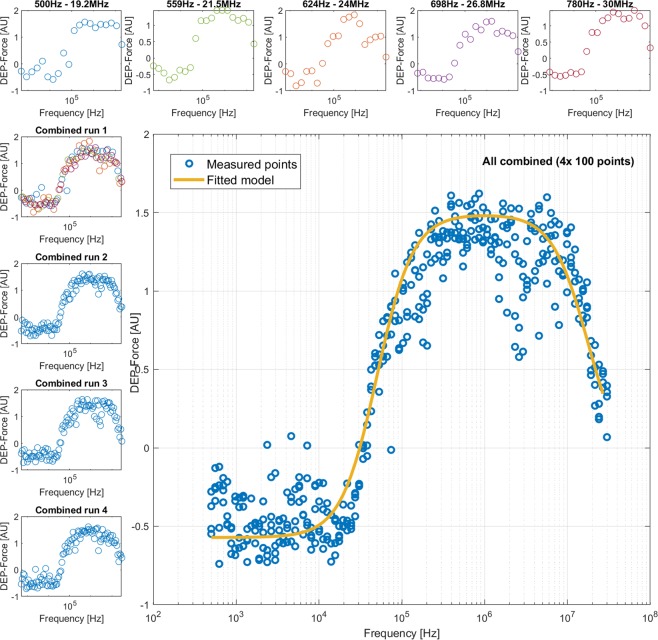
In order to provide ultra-high-resolution (100-point) DEP spectra, K452 cells were analysed by 20-point spectra (top row) that, when combined, provided a single spectrum with 100 equally-spaced points (shown here as Combined run 1). Three more 100-point spectra (Combined runs 2–4) were performed separately; these were then combined into a single 400-point spectrum (main panel). Best-fit models were generated for the 400-point spectrum (shown) and compared to best-fit models for 100-point and 20-point spectra in order to determine whether 20 points is as accurate as higher-resolution spectra for robust data extraction.

The DEP system uses 20 wells, enabling 4 decades to be studied at approximately 5 points per decade. Given that dielectric dispersions in the DEP spectrum take approximately one decade to complete^16^, we can assume that 20 points affords sufficient resolution to accurately observe all of the key features of the DEP spectrum (low, middle and high frequency plateaus and two dispersions). However, since the acquisition of a 20-point spectrum takes such a short time, we were able to examine this assumption by rapidly acquiring 100-point spectra in a little more than five minutes. This was achieved by analyzing five chips in rapid succession, each loaded with the same cell solution but energized with a set of frequencies shifted slightly across the logarithmic scale with respect to those used on the previous chip. Using five chips and shifting the frequency range of each successive chip by a factor of 1.118, we synthesized the equivalent of 100-point spectra, as shown in Fig. 4. We acquired and modelled 100-point spectra from K562 cells, RBCs and yeast cells.

This allowed us to test the hypothesis that 20 points are sufficient to produce accurate data for modelling. The five 20-point spectra were modelled separately, then combined to produce a 100-point spectrum that was remodeled. One-way ANOVA with multiple comparisons across all frequencies showed that none of the data points was statistically different from the model mean (p > 0.05 for all points). We also analyzed the statistical distribution of each frequency of ten 100-point spectra to examine data point distribution and significance. A D’Agostino and Pearson omnibus normality test was conducted across all 100 frequencies, which revealed that all but three frequencies demonstrated normal distribution across the 10 repeats.

### Benchmarking against published data

We compared the electrophysiological data acquired using the 3DEP platform with literature values reported either by DEP or patch clamp. For DEP measurement, we compared values acquired for Jurkat cells, which have been performed by a relatively large number of other practitioners; for patch clamp we compared RBCs, platelets, Jurkats, K562 and HL-1 cells. Note that additionally, the cytoplasm conductivity of HL-1 cardiomyocytes has previously been benchmarked against standard electrophysiological methods used in cardiac physiology^18^.

#### Bench marking DEP parameters

**Figure 5:**
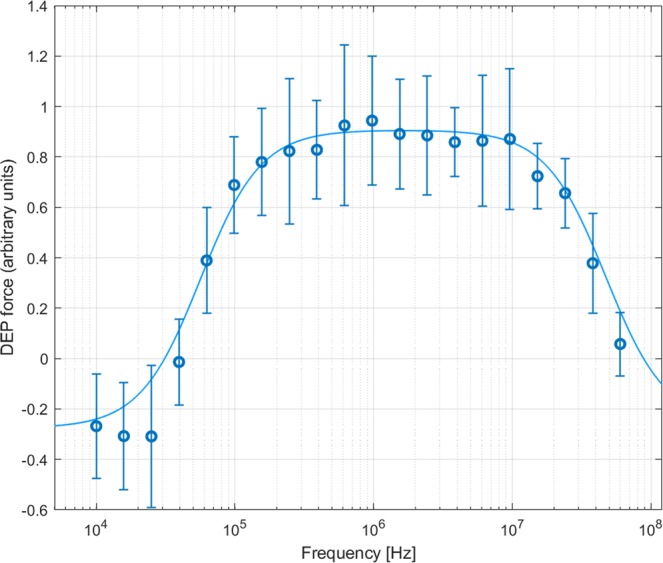
Averaged DEP spectra of Jurkat cells based on fifteen 10-second measurements. Points indicate mean measurements of change in light intensity passing through the wells after 10 seconds, scaled to a best-fit curve (line) indicating the best-fit Clausius-Mossotti factor for two populations (see text). The correlation coefficient between mean data and best-fit curve was r^2^ = 0.998.

To compare with existing DEP data, 3DEP spectra of Jurkat cells were acquired (n = 15), Fig. 5. When modelled against a multi-population model^16,22^, it was found that within the average population 90% of Jurkat cells conformed to a dielectric parameter set resembling a healthy cell population, whose extracted data closely follow data reported by other impedance-based methods, including dielectrophoresis, reported in the literature^36–41^ (Table 1). The additional 10% of the population resembled the majority population but had significantly lower cytoplasm conductivity (70 mSm^−1^, a reduction of 85%) and membrane conductance (by 50%), potentially indicating either cell damage or apoptosis caused by stresses during sample preparation. The only difference of note between the electrical properties of the “healthy” cell population and Jurkat data acquired by other researchers was the lower value of membrane conductance using the 3DEP system, most likely due to alterations in medium composition, as the inclusion of MgCl_2_ and CaCl_2_ in the medium has been shown to prevent ion leakage in low conductivity media^18^.

**Table 1:** Best-fit dielectric properties of Jurkat cells determined by the DEP well system, compared to published values.

	3DEP	Literature
Membrane capacitance (mF/m^2^)	9.96	13.24^A^; 13.5–14^B^; 7.1^C^; 7.6–10^D^, 8.3^E^
Membrane conductance (S/m^2^)	25	35–82^A^; 137^C^; 75^E^
Radius (measured optically, µm)	6.5 ± 0.9	5.25^A^; 7.4^B^; 7.6^C^; 5.18^D^; 7.4^E^; 5.5^F^
Cytoplasm relative permittivity	60	90^A^; 80^E^; 60^F^
Cytoplasm conductivity (S/m)	0.48	0.7^A^; 0.84–1.1^B^; 0.25^D^; 0.15^E^; 0.3^F^

Relative permittivity is the ratio of the permittivity of a substance to the permittivity of free space or vacuum. Sources: ^A^Pethig and Talary^36^; ^B^Kiesel *et al*.^37^; Sukhorukov *et al*.^38^; ^D^Garner *et al*.^39^; ^E^Reuss *et al*.^40^; ^F^Zhang *et al*.^41^.

#### Whole-Cell membrane capacitance

We also compared the values of membrane capacitance and conductance acquired by DEP with those acquired by the gold-standard patch clamp method, which produces values of whole-cell capacitance and conductance. We were able to produce comparable data from DEP spectra (which produces values per unit area) by multiplying the derived values of specific membrane capacitance and conductance with the average surface area calculated from acquired radius measurements (n > 100); though the particles were not spherical, using a spherical assumption for both model and assumed area produces highly comparable data.

Comparative data for membrane capacitance are summarised in Table 2. Considering first the analysis of human RBCs; these highly regular cells provide a useful technical standard due to their highly homogeneous and well-characterised morphology. RBCs have a known surface area and membrane permittivity, which together give a theoretical value of 1.3–1.6 pF^42^ and values showing this have been reported^43^. RBCs fractioned from whole blood, and whole blood itself were analysed in this study. Using a mean measured RBC radius of 4.1 ± 0.1 µm, the mean whole-cell capacitance of isolated RBCs (n = 185 experiments) was 1.32 ± 0.02 pF. For platelets (mean radius 0.51 ± 0.04 µm) isolated from whole blood samples, a whole-cell capacitance of 121 ± 7 fF (n = 5) was calculated. This is consistent with the literature value, 128 ± 8 fF, cited in electrophysiological studies^44,45^. For Jurkat cells (n = 15), the mean value of whole-cell capacitance of 5.3 ± 1.4 pF acquired by DEP was smaller than published values obtained from patch clamping^46^ of 7.5 ± 0.8 pF. HL-1 cardiomyocytes (n = 5) were measured at 7.9 ± 1.4 pF, approximately 20% smaller than those obtained by patch clamp on the same cells in an attached state^47^ (9.9 ± 0.9 pF), whilst K562 cells (n = 12) were measured at 4.0 ± 0.5 pF, which is comparable to the lower end of a range of published values^48^.

**Table 2:** Best-fit values of whole-cell capacitance from DEP data, compared to published data derived from patch clamp studies.

Cell type	DEP (pF)	Literature (pF)
RBC	1.32 ± 0.02	1.3–1.6^A,B^
Platelets	0.121 ± 0.007	0.128 ± 0.008^C,D^
Jurkats	5.3 ± 1.4	7.5 ± 0.8^E^
HL-1	7.9 ± 1.4	9.9 ± 0.9^F^
K562	4 ± 0.5	4–7^G^

Sources: ^A^Rodighiero *et al*.^42^; ^B^Browning *et al*.^43^; ^C^Maruyama *et al*.^44^; ^D^Tolhurst *et al*.^45^; ^E^Ross *et al*.^46^; ^F^Hansen *et al*.^47^; ^G^Smith *et al*.^48^.

It is notable that whilst cells that are very regular in size show very little difference between patch clamp and DEP, with cells that exhibit greater variation in size DEP consistently produced smaller values of membrane capacitance than patch clamp. There are a number of potential reasons for this discrepancy; for example, it may be that adherent cells have lower capacitance in suspension, However, anecdotal evidence from patch clamp practitioners suggests another possible cause. Given the very small numbers of cells typically presented in patch clamp research (four in the case of the Jurkat data cited here) and the difficulty in performing patch-clamp experiments, there exists a pragmatic tendency to select the largest, easiest cells to analyse. Since whole-cell capacitance is related to cell surface area, this would lead to a systematic bias towards larger values; for example, selecting cells with radii around 20% larger than the mean would account for the shift seen in the data from Jurkat cells. Given the small numbers of cells typically analysed, it cannot be assumed that these are representative samples of the cells under scrutiny. DEP, on the other hand, analyses much larger numbers of cells DEP (our Jurkat data were based on ensemble measurements of *circa* 300,000 cells) and measures all cells in the sample, independent of their size, is thus far less prone to such bias.

#### Whole-Cell membrane conductance

Whilst the application of DEP for measurement of membrane capacitance is straightforward and accurate, the case for membrane conductance is more complex. When a membrane potential measurement is made using whole-cell patch clamp, it corresponds to the flow of current passing from the cell interior to the exterior – that is, the transmembrane conductance. DEP conductance accounts for both this characteristic and a second component of charge movement tangentially *across* the membrane. This latter effect has previously been attributed to conduction due to surface charge, specifically charge in the electrical double layer attracted to charges on the cell surface^43^. It has been suggested that this surface component could be significantly larger than the conventional whole-cell parameter, particularly for smaller cells, making it difficult to extract the membrane conductance parameter. However, this can still be used as an effective discriminator of cellular properties and has been used to detect changes in RBC membrane properties due to malarial infection^49^, as well as identifying circadian rhythms in RBC electrophysiology^25,26^. For Jurkat cells a value of 11.3 nS (equivalent to a specific membrane resistance of 0.45 kΩ.cm^2^) was obtained which is larger than common values of whole-cell conductance reported for other cell types of similar origin by patch-clamp, though by less than an order of magnitude. However, RBCs exhibited a range of values centered on 201 nS, approximately one thousand-times larger than quoted values such as 442 pS by Duranton *et al*.^50^. The size of increase is similar to that reported by other researchers^43^, indicating that this result is not an instrument-generated artefact. Interestingly, the membrane conductance of platelets was found to be even higher, at 365 nS, suggesting a size-related conductance similar to that observed in nanoparticles^51^. Previous work in DEP has indicated the usefulness of DEP-derived membrane conductance as a stand-alone biomarker, but whilst the parameter clearly has significant potential, further exploration is required to fully unlock its electrophysiological significance.

### Measurement near physiological ionic strength

**Figure 6:**
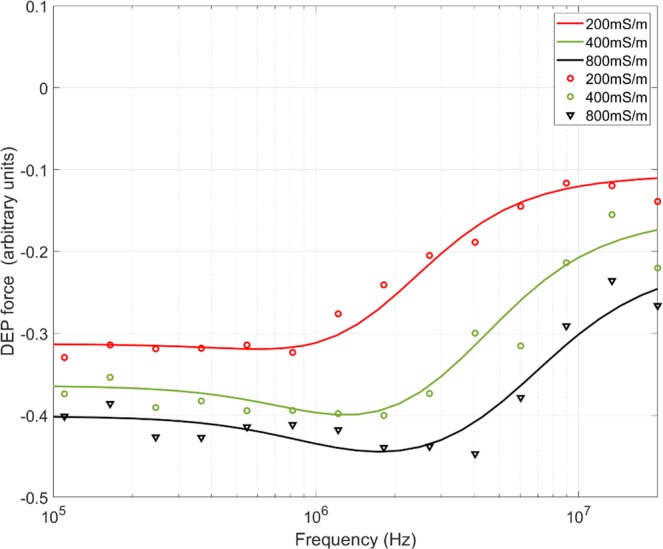
Platelets in high-conductivity media. DEP spectra of human platelets in high-conductivity media, ranging from 200 mS.m^−1^ to 800 mS.m^−1^.

A significant limitation of most DEP measurement approaches is that the use of microfabricated planar electrodes precludes the use of high ionic strength media (typical physiological media have conductivity ca. 1400 mS.m^−1^); the thin gold films used for DEP are easily damaged by the current densities caused by the high field strengths, leading to electrolysis or “burning” of the electrodes. As a consequence, media used in DEP systems are often limited to about 100 mSm^−1^. The DEP-Well approach instead uses relatively thick layers of copper, and avoids the high charge concentrations associated with thin-film fabrication; as a consequence, it is possible to use media of considerably higher conductivity. We were able to obtain robust data at considerably higher medium conductivities than are conventionally analysed. For example, the results of DEP analysis of platelets suspended in 200, 400 and 800 mSm^−1^ solutions are shown in Fig. 6. Even at very high medium conductivity it was possible to acquire largely noise-free spectra; modelling the data showed steady elevation of cytoplasm conductivity, from 150 mSm^−1^ in the lowest conductivity solution to 250 mS.m^*−*1^ in the intermediate and 400 mS.m^−1^ in the highest, suggesting that platelets are susceptible to changes in the conductivity of their suspending medium. However, variation of medium conductivity did not affect either the membrane capacitance or conductance, which remained at 7.6 mF.m^−2^ and 35.7 kS.m^−2^ respectively.

### Recovery and culturing of cells after analysis

An important advantage of DEP over many other measurement techniques is that cell viability is largely unaffected by low electric field exposure on short timescales^52,53,54^. In order to assess whether we could extract and culture viable cells after analysis, we performed DEP measurements on HeLa cells (n = 15) in autoclaved chips, the spectra of which can be found elsewhere^22^. We then removed cells carefully from the chips with a pipette, and placed them in the well of a 96-well plate. The average volume recovery of the 75 µl sample was found to be 58 ± 4 µl, suggesting a recovery rate of 77% assuming cells remained equally distributed throughout the chip. Cells were monitored for viability over several days and at least one passage; 24 hr after extraction, cell viability by Trypan Blue exclusion was assessed at 91 ± 4%. After 72 hrs viability was slightly lower at 88 ± 5%, and post-passage remained consistent at 90 ± 4%.

### Humane detection of ultradian rhythms in vole red blood cell electrophysiology

The simplicity and rapidity of the measurement system, together with the relatively low cell numbers required for each experiment (a total of ca. 10^6^ are required to fill the chip) allows the observation of phenomena that simply cannot be observed by other methods. One such example is the observation of diurnal rhythms in electrophysiology; recent work by Henslee *et al*.^25^ showed that 3DEP analysis can be used to detect circadian rhythms in the electrophysiology of human red blood cells. Analysis of the electrophysiological properties of cells, when correlated with other techniques (at much lower sampling rates) such as inductively coupled plasma mass spectrometry and flow cytometry suggested that these circadian rhythms are associated with transmembrane K^+^ transport.

An advantage of the 3DEP system is that chip loading is simple, and as cell requirements are relatively small, the system lends itself well to rapid blood testing; 1 µl typically contains >10^6^ red blood cells and >10^3^ white blood cells. 10 µl diluted into 5 ml of 10 mS.m^−1^ DEP medium gives sufficient cell density for analysis by 3DEP, with minimal impact on medium conductivity (raising it by ca. 1.5 mSm^−1^). This makes it useful for rapid testing of blood samples from humans or animals.

By way of demonstration, we investigated biological rhythms in blood samples taken from voles. Animals such as voles express “ultradian” biological rhythms far faster than human circadian rhythms, typically lasting only 2 hours. Conventional methods of analysis^35^ require at least 1 ml of blood for analysis. Since this represents a sizable fraction of the blood of small rodents, every measurement requires the sacrifice of the animal; robust measurement of transient phenomena might require the sacrifice of 70–100 animals^42,55^. This impacts on the ability to compare systemic rhythmicity in human and rodents, because unlike human studies, rodent studies do not permit within-individual analysis^56^. Conversely, 10 µl can easily be acquired using a pinprick or small nick to the tail, and obviates the need to sacrifice the animal. Furthermore, the same animal can be used in longitudinal studies, significantly increasing robustness of data by allowing multiple experiments to be performed on the same animals.

**Figure 7:**
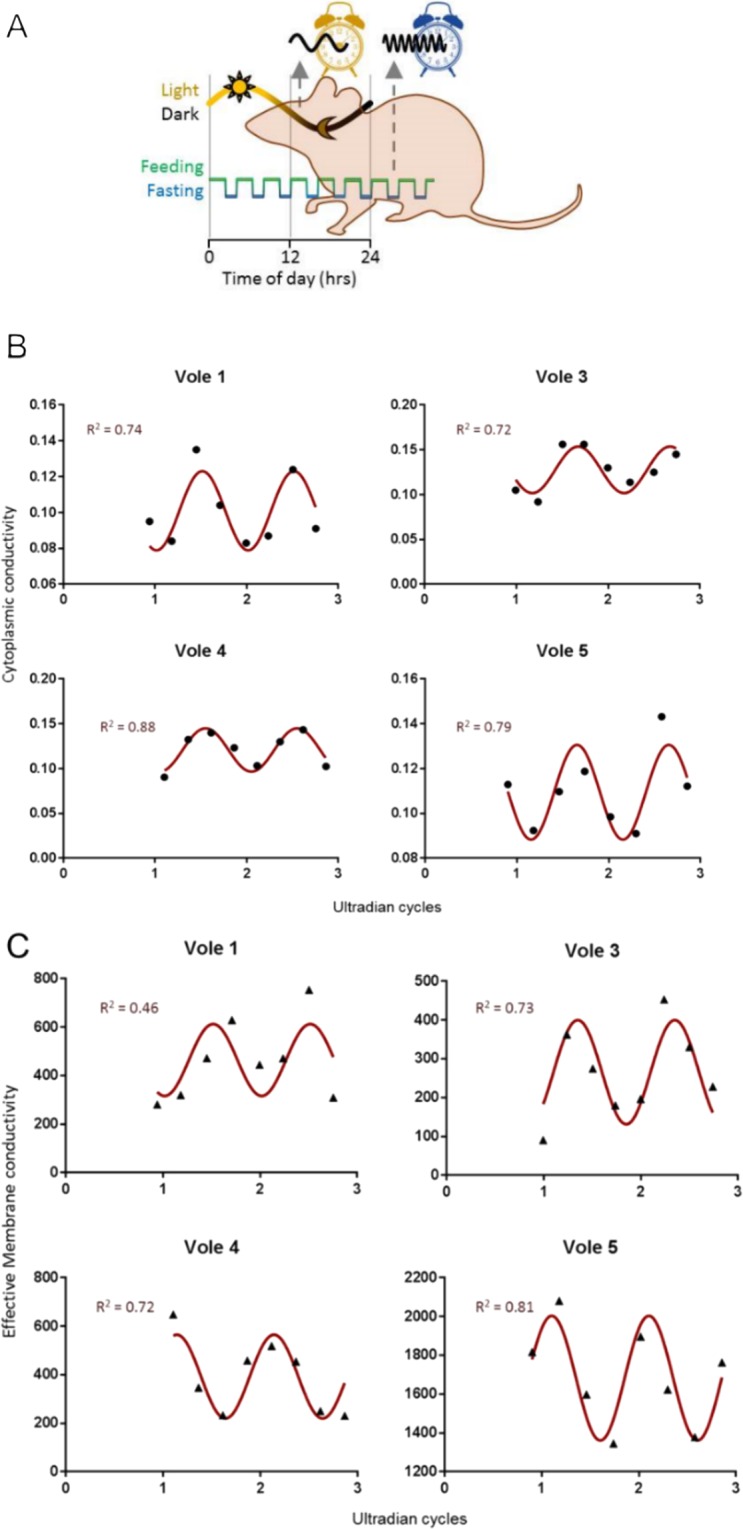
Whole blood assay. (**A**) In nature and the laboratory, the common vole expresses short (ultradian) feeding-fasting and sleep-wake cycles, whilst being under a circadian light-dark cycle. (**B**) DEP measures of cytoplasmic conductivity fits to a series of consecutive vole blood sample for 4 voles, fitted with a cosinor curve. Data points (black circles) are mean parameters determined by at least n = 3 spectra fits at each time point. R^2^ values indicate the goodness of fit to the cosinor curve determined by the phase of observed activity (actogram) and fitted amplitude and mesor (mean estimating statistic of rhythm) to DEP data (red line). (**C**) DEP measures of effective membrane conductivity fits to a series of consecutive vole blood sample for 4 voles, fitted with a cosinor curve. Data points (black circles) are mean parameters determined by at least n = 3 spectra fits at each time points. r^2^ values indicate the goodness-of-fit to the cosinor curve determined by the phase of the actogram and fitted amplitude and mesor to DEP data (red line).

To explore the advantages of this approach, we studied blood samples acquired by tail nick from four voles (*Microtus arvalis*) every 30 minutes for 4 hours. The results can be seen in Fig. 7. For each sample, cytoplasmic conductivity and specific membrane conductance were determined; a cosinor was fitted all samples to determine if a diurnal rhythm was present. Parameters were fitted with a non-linear r^2^ between 0.72–0.88 in all cases for cytoplasm conductivity and all but one for membrane conductance. The periods of these cosinor fits were fixed to the behavioural ultradian period (1.47–1.55 hours), taken from the observed behaviour activity in voles. Interestingly, in the three samples where both membrane conductance and cytoplasmic conductivity were successfully fitted, they showed the same counterphase relationship observed in human blood over the circadian cycle^25^. This is a unique observation given that, in contrast to slow circadian rhythms, no physiological measures exist to track rapid ultradian rhythms in blood.

As well as allowing for rapid, humane electrophysiology, this work has the potential to be translated to human studies, with implications in the broader chronobiology field, particularly in relation to determining the internal circadian phase of a human subject. Unlike melatonin, which is detected by radioimmunoassay, DEP assays require only a pinprick of blood (similar to that used in diabetes assays), are performed in minutes, and our observations here and in Henslee *et al*.^21^ suggest it is possible they can be performed reliably using whole blood sampled from animals and humans. By comparing single-point measurements of membrane conductance and cytoplasmic conductivity with a known standard curve of their circadian variation across a population, it may be possible to rapidly determine and individual’s circadian phase using DEP.

## Conclusion

The 3DEP system delivers significant time savings and higher sample throughput, whilst performing favourably when compared with other electrophysiological methods such as the ‘gold-standard’ patch-clamp method. For phenotypically homogeneous cells, our capacitance values match exactly with both published and theoretical data. For more heterogeneously-sized cells, DEP mean values tend to be slightly lower than published data - most likely because DEP samples a much larger cell population and is therefore far less susceptible to type 1 errors resulting from small sample sizes. Our system works well for particles which are difficult to patch due to their small size, whilst rapidity of measurement allows time-dependent changes in cell properties to be measured with greater accuracy.

## Data Availability

The datasets generated during and/or analysed during the current study are available from the corresponding author on reasonable request.
